# Association of Mortality-Related Risk Factors in Patients with COVID-19: A Retrospective Cohort Study

**DOI:** 10.3390/healthcare9111468

**Published:** 2021-10-29

**Authors:** Shazia Rehman, Nadia Rehman, Ayesha Mumtaz, Jindong Jiang

**Affiliations:** 1 Department of Biomedical Sciences, Pak-Austria Fachhochschule, Institute of Applied Sciences and Technology, Haripur 22621, Pakistan; rehmanshazia.malik@gmail.com; 2 Department of Mathematics, Wah Campus, COMSATS University Islamabad, Wah Cantt 47040, Pakistan; nadia_rehman@ciitwah.edu.pk; 3 College of Public Administration, Zhejiang University, Hangzhou 310058, China; 4 Department of Psychology, Jinhenyi School of Education, Hangzhou Normal University, Hangzhou 310023, China

**Keywords:** COVID-19, SARS-CoV-2, risk-factors, in-hospital mortality, public health, epidemiology

## Abstract

COVID-19 is a rapidly disseminating infectious disease conferred by the World Health Organization (WHO) as severe acute respiratory syndrome coronavirus 2 (SARS-CoV-2). Epidemiological and clinical characteristics data have been identified for patients with COVID-19, but mortality-related risk factors and a comprehensive clinical course of disease in a developing country have not been specifically defined. This retrospective, single-center cohort study involved all successive inpatients having a positive COVID-19 polymerase chain reaction (PCR), with deceased or discharged clinical outcomes from 1 January to 10 May 2021. Data were extracted from electronic medical records on demographic, clinical, radiological, and laboratory findings as well as complications faced and treatment provided during follow-up, involving serial samples for viral RNA identification, and compared between the dead and survivors. To investigate the risk factors associated with in-hospital mortality, we employed the multivariate logistic regression model. In this study, 2048 patients were involved, 1458 of whom were discharged, and 590 died in hospital. More than half of patients were identified as male with old age being the potential risk factor of mortality. Exactly 94.8% of all patients presented with fever at the time of admission. Several comorbidities were present in the study population, with the most frequent comorbidity being cardiovascular diseases (1177 of 2048) and hypertension (975 of 2048) followed by cerebrovascular disease and diabetes mellitus. Mortality rates for infected patients were observed as higher in severe patients (46.3%) compared with non-severe cases (26.1%) during a follow-up. Multivariate regression analysis showed a significant association of in-hospital mortality of patients with older age, presence of hypertension and cardiovascular diseases as underlying comorbidities, increased level of cardiac troponin I and d-dimer concentration on admission, as well as septicemia and ARDS as a complication during illness. To minimize the risk of death in COVID19 patients, as well as the risk of severe complications, urgent public health measures should be properly planned and implemented on those vulnerable populations. To detect early manifestations of clinical problems, thorough and regular follow-up is warranted.

## 1. Introduction

At the beginning of 2020, the outbreak of severe acute respiratory syndrome coronavirus 2 (SARS-CoV-2) infection triggered a disease pandemic named COVID-19 [1,2]. There is virtually no country in the world not affected by COVID-19, and despite this immense burden, healthcare services across the globe have never operated more efficiently. There have been tens of thousands of newly infectious patients, and thousands of deaths were registered every single day [3,4]. It all began in Wuhan, China, then spread exponentially across the globe. After China, the core of the pandemic moved into Europe and the United States. The World Health Organization (WHO) declared COVID-19 a pandemic on 11 March 2020 because of its unprecedented rates of dissemination, intensity, and inaction [5,6]. COVID-19 is triggered by SARS-CoV-2, which is genetically widely divergent enough from the strongly connected SARS-CoV to be deemed a novel human-infecting beta coronavirus. It primarily affects the respiratory system, and the severity varies from asymptomatic or moderate to serious to critical illness. Even though the present estimation of the case fatality rate of COVID-19 is lower than 5%, up to 15–18% of patients might get severe or critical illnesses, some of them requiring ICU care and mechanical ventilation. The current novel coronavirus disease has broad spectrums of clinical features varying from asymptomatic disease carriers to respiratory collapse manifestations seeking mechanical ventilation support to ICU (intensive care unit), and multi-organ and systemic symptoms ranging from sepsis to multiple organ dysfunction syndromes (MODS). Due to its severity, manifestations of coronavirus of a mild category are encountered by the majority with no or mild pneumonia effects, moderate and severe with sepsis, MODS, and severe pneumonia [7].

Comprehensive knowledge of the disease is however inadequate and emerging as COVID-19 is a new disease. While many case studies have been published, at the time of publishing several patients in those studies were hospitalized. However, to our knowledge, there have been no systematic prior trials of patients with definitive outcomes. Therefore, the assessment of risk factors for serious illness and mortality in these earlier case reports is not very reliable. Besides that, specifics of the clinical and virologic course of the disease have not been adequately established so far. Consequently, this research work aimed to identify and correlate the epidemiological, demographic, clinical, laboratory, and radiological characteristics, and severity assessment on admission as well as the complications in the Pakistani setting. Potential mortality-related risk factors for COVID-19 patients were examined to provide clinical evidence for a deeper understanding of the infected cases and to prevent death.

## 2. Materials and Methods

This section comprises detailed information and procedure involved in the collection of data samples for COVID-19 patients. In addition, univariate analysis and a multivariate logistic regression model are addressed to determine the relationship of possible risk factors associated with SARS-CoV-2 infected patients.

### 2.1. Data Source and Study Population

A retrospective single cohort study was carried out in the Pakistan Institute of Medical Sciences (PIMS), Islamabad, the capital of Pakistan during the third and fourth wave of the pandemic. All consecutive patients admitted with confirmed COVID-19 in a designated tertiary hospital from January 1 to May 10 were included. The final date of follow-up was 20 June 2021. Written or oral informed consent was obtained from patients. A COVID-19 case report form was designed to document primary data regarding patient demographic and clinical characteristics and laboratory and radiological findings as well as the complications and treatment given during hospitalization from medical records. The following information was extracted from each patient: age, gender, medical history, COVID-19-related exposure history, co-morbidities, symptoms, severity assessment on admission, laboratory findings, imaging features, treatment is given, and in-hospital death as the outcome variable. A confirmed COVID-19 case has been described as a positive result of an RT-PCR test on nasal and oropharyngeal swab specimens in real-time. All healthcare professionals caring for infectious patients have been professionally qualified to incorporate infection prevention strategies and protocols with proven experience.

**Figure 1:**
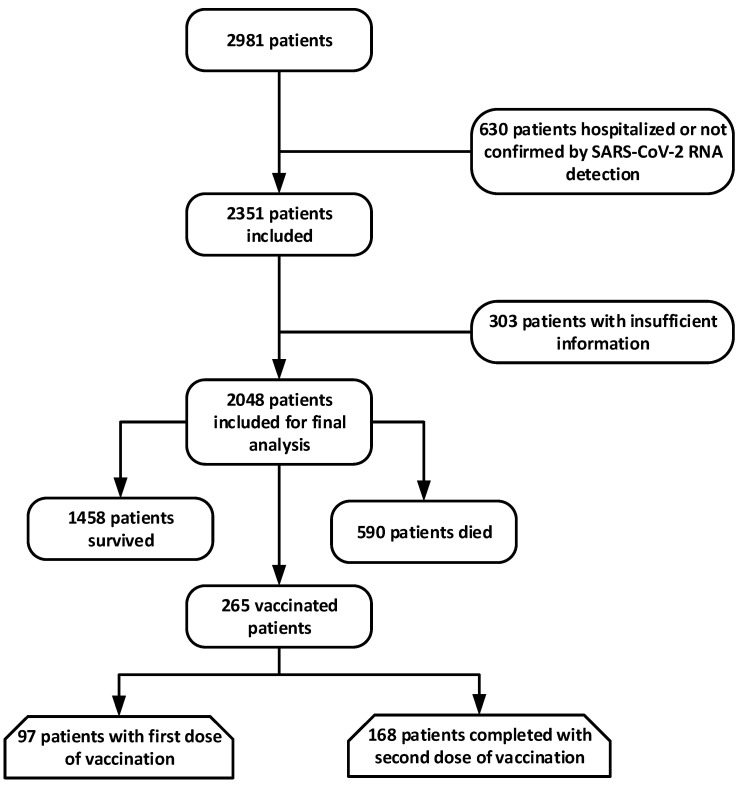
Patient’s selection flow chart.

The study population screening criteria were based on the fact that any patient who had a recent onset of fever, fatigue, cough, and/or body aches was included. The history of exposure has been described as exposure to people with SARS-CoV-2 confirmed infection. On admission, serum cytokine (IL-6, IL-8, and TNF-α) levels were assessed. In compliance with the International Classification of Diseases (revision 10), the existence of underlying co-morbidities was established. All cases of COVID-19 in this research have been reported based on interim guidance from the WHO. The patient’s selection flowchart is presented in Figure 1.

Until final data entry, patient data were reviewed by two qualified biostatisticians for consistency and then placed in a computerized database. The clinical outcomes were listed as in-hospitalization, recovered and discharge from hospital, and in-hospital death. Severe COVID-19 was identified by the Infectious Diseases Society of America and the American Thoracic Society for Diagnosis and Treatment of Adults with Community-acquired Pneumonia in 2019 [8]. Ethical approval for the study was obtained from the Ethics Review Committee of the PIMS hospital regulatory body Ministry of Health Pakistan. Approval reference: (KIIT 2021/PK 2021–36- MS 287).

### 2.2. Statistical Analysis

The basic demographic and clinical aspects of patients with associated co-morbidities, complications, and treatment of hospitalization have been summarized in frequencies and percentages for the selected population. The descriptive statistics for continuous data are median and interquartile ranges. For continuous variables, Mann–Whitney U testing was done and the χ2 test and the Fisher exact test were employed as appropriate for categorical variables. In addition, the in-hospital mortality rate was also computed for each reference category, as the outcome could also be impacted by patient characteristics. We performed univariate analysis to examine the factors associated with the death of patients from COVID-19. The significant variables determined by the univariate analysis (*p* < 0.05) were brought into the multivariate analysis, in which those variables were adjusted. A value of *p* less than 5% has been considered statistically significant. All statistical computations were conducted using the SAS software, version 9.3 (SAS Institute Inc., Cary, NC, USA) unless otherwise mentioned.

## 3. Results

This section provides a comprehensive discussion on the results obtained from univariate and multivariate analysis in which epidemiologic, demographic, and clinical findings of infected patients have been discussed. The complications faced during illness and treatment provided with clinical outcomes have been described in detail. Finally, the possible risk factors associated with in-hospital death have been demonstrated for a deeper understanding. This investigation is principally significant for policymakers and health practitioners to better understand the mortality-related risk factors of COVID-19 patients.

### 3.1. Epidemiologic, Demographic, and Clinical Characteristics

The epidemiologic and demographic characteristics of patients with COVID-19 are presented in Table 1. A total of 2981 patients were hospitalized in PIMS hospital with COVID-19 from 1 January 2021 to 10 May 2021. After dropping 630 patients who were either hospitalized or not confirmed by SARS-CoV-2 RNA detection as of 10 May 2021 and 303 inpatients without the most relevant information present in their medical records, we eventually included 2048 inpatients for the final analysis. Almost 590 patients died during hospitalization, and 1458 were recovered and discharged. The median age of the study population was 56 years ranging from 18 years to 88 years, of whom 1681 (82.1%) were aged sixty-one years or older. A higher proportion of death was observed in patients aged sixty-one or above. More than half of patients (59.4%, 1217 of 2048) were identified as male with a higher proportion of deaths (415 of 590) than female patients (175 of 590). Among the deceased, 203 out of 590 were identified as obese with a BMI ≥ of 30. A total of 522 out of 2048 patients were identified as smokers and the mortality rate was 18% among non-survivors. About 32.8% of patients with deceased cases had an exposure history. Exactly 229 patients were diagnosed by computed tomography (CT) screening and were asymptomatic. Surprisingly, among 2048 infected patients, 265 patients were vaccinated. Among those, 97 had received their first dose of vaccination and the remaining had completed their second dose. Deceased cases endured considerably longer periods from the onset of infection to the outpatient visit and longer periods from the onset of infection to hospital admission compared to the cases discharged. Most patients reported at least 1 of the following symptoms in the study population: fever (94.8%), cough (72.6%), fatigue (57.5%), sore throat (57.0%), chest pain (54.7%), nasal congestion/rhinorrhea (50.5%), dyspnea (46.2%), chill (46.2%), dizziness and headache (33.5%), urinary incontinency (30.6%), chest distress (29.2%), abdominal pain (20%), Myalgia or arthralgia (15.1%), diarrhea (9.9%), nausea and vomiting (6.1%). Fever (85.1%) and cough (72%) was found the most common risk factor among deceased patients with COVID-19 followed by fatigue (67.1%) and sore throat (65.4%). A range of comorbidities was present in the study population, with the most prevalent comorbidities being CVD (1177 of 2048) and hypertension (975 of 2048) followed by cerebrovascular disease and DM (Table 1). Deceased cases had a higher proportion of hypertension (75.3%, 444 of 590) and diabetes mellitus (66.9%, 395 of 590). Overall, 154 cases of COPD (7.5%) and 135 cases of asthma (6.6%) were reported. Among 2048 cases, the proportion of severe cases was identified as 1411 (68.9%), whereas 31.1% were non-severe at the time of admission. Mortality rates for infected patients were observed as higher in severe patients (46.3%) compared with non-severe cases (26.1%) during follow-up.

### 3.2. Laboratory Indicators and Imaging Features

Table 2 presents the results of radiologic and laboratory results of the study population. On admission, 57.1% of all patients had lymphopenia and 34% of patients had thrombocytopenia. Among 2048 patients, 655 had an abnormality of neutrophils. Compared with survivors, inflammatory biomarkers such as PCT (52.5%), ESR (83.6%), and serum ferritin (42.7%) were significantly higher in deceased cases, except CRP, which was comparatively lower when compared with survivors. The elevated levels of albumin (47.6%), globulin (42%), ALT (33.5%), AST (30.7%), D-dimmer (47.6%), and creatinine (25.5%) were observed during laboratory investigation of patients with COVID-19. Serum cytokine levels of IL-8 and TNF- α were exceptionally higher in deceased patients than in survivors, except for IL-6. Proteinuria occurred in 1575 (76.9%) patients. Among deceased cases, 11.5% were identified with prolonged thrombin time. Compared with survivors, cardiac biomarkers such as troponin I (93.2%; 550 of 590), CK (27.9%; 165 of 590), and lactate dehydrogenase (67.3%; 397 of 590) were significantly higher in deceased cases. Moreover, deceased cases had a higher incidence of bilateral pneumonia (80.3%) and pleural effusion (67.2%).

### 3.3. Complications and Treatment during Hospitalization

Table 3 represents the complications and treatments given to COVID-19 patients during hospitalization. The complications of COVID-19 were assessed during the follow-up phase. The most frequently observed complication was septicemia (51.9%; 1062 of 2048), which occurred approximately in half of the patients, followed by ARDS and respiratory failure. However, higher mortality was observed in patients with ARDS (98%; 578 of 590). The rest of the complications included acute kidney injury (17.9%), cardiac injury (19.3%), liver dysfunction (25.5%), coagulopathy (19.8%), bacteremia (17.5%), and hyperglycemia (30.7%). The frequency of complications was higher in expired cases as compared with those who recovered and were discharged (all *p* < 0.05).

During hospitalization, antibiotics and antiviral medications were used primarily to treat COVID-19. Among 2048 patients, 1970 received antibiotics, and 502 received antivirals. In deceased cases, antiviral drug use was more prominent than in survivors. The use of intravenous corticosteroids and immunoglobulin differ substantially between the survivors and those who died. Of the 2048 patients, 395 received high flow oxygen therapy, and 377 received nasal cannula or mask. Precisely 291 patients required noninvasive mechanical ventilation, of whom 262 (44.4%) died. Among 2048 patients, Invasive mechanical ventilation occurred in 737 patients and only 165 patients survived. A total of 68 patients were treated with extracorporeal membrane oxygenation (ECMO), of whom only 15 survived. Exactly 174 patients underwent renal replacement therapy and, out of that, 159 died.

### 3.4. Potential Risk Factors Associated with In-Hospital Mortality

Table 4 summarizes the results of risk factors associated with in-hospital death of patients with SARS-CoV-2 infection. Twenty significant variables from univariate analysis were put into multivariate analysis to identify reliable predictive variables for in-hospital mortality. Seven variables were observed as significantly associated with the in-hospital death of infected patients. We found that older age was a potential factor for a higher risk of mortality in infected cases, whereas the presence of hypertension and CVDs as underlying comorbidities have a significant impact on increased mortality of COVID-19 patients. Increased levels of cardiac troponin I and d-dimer on admission were observed as significant for those who died. The occurrence of sepsis and ARDS during hospitalization were significant risk factors associated with in-hospital mortality of patients infected from SARS-CoV-2.

## 4. Discussion

This retrospective cohort study presented detailed statistics on the epidemiologic, demographic, therapeutic, laboratory, and radiological features as well as the complications, treatment, and severity assessment on the admission of discharged and deceased patients of COVID-19 in Islamabad, Pakistan. In this study, multiple risk factors were reported, consistent with some past studies, associated with in-hospital mortality of COVID-19 patients. In particular, old age, hypertension, pre-existing CVD, increased levels of cardiac troponin I and d-dimer concentration on admission, septicemia, and ARDS were observed as significantly associated with a higher proportion of in-hospital deaths. Additionally, DM, elevated levels of blood IL-6, LDH, and high lymphocyte count were more commonly seen in those who died of COVID-19 illness. Almost 68.9% of patients in this study were classified as severe cases, which could vary from the findings of the previous studies.

The proportion of elderly (aged ≥ 70) and males in our study sample was relatively high compared with some recent studies. Old age has previously been identified as a significant independent mortality indicator for COVID-19 patients [9,10]. Likewise, researchers have also indicated that males have a greater frequency of getting infected from SARS-CoV-2 [11,12]. In this study, the majority of elderly males were considerably related to death in COVID-19 patients. Elderly patients are known to have weakened immune systems and are thus more vulnerable to ARDS [12]. Hence, it can also be shown why elderly male patients are harder to treat and can potentially lead to poorer outcomes. However, hypertension, DM, and pre-existing cardiovascular conditions were the most important comorbidities observed in our research, aligned with those in previous researches [13,14,15]. These conditions were higher, particularly in deceased COVID-19 cases. These comorbidities are frequently treated with angiotensin-converting enzyme (ACE) inhibitors and angiotensin II type I receptor blockers (ARBs), leading to up-regulation of ACE2 [16,17]. Consequently, the higher concentration of ACE2 may partially describe the higher prevalence of severe COVID-19 in hypertension patients [18]. We recommend that patients with cardiac diseases, hypertension, or diabetes that are treated with ACE2-increasing drugs may be at higher risk for severe COVID-19 infection and that, therefore, ACE2-modulating therapies like ACE inhibitors or ARBs should be supervised attentively.

In certain clinical forms, COVID-19 was found similar to SARS and MERS. Fever, cough, and myalgia, accompanied by chest pain and shortness of breath, were the most frequent symptoms of COVID-19 patients, although the signs of the upper respiratory tract and gastrointestinal symptoms were comparatively low. In the present investigation, fever occurred in about 95% of patients compared to 98%–100% of patients with SARS or MERS [19,20]. In the current analysis, approximately 5% of patients who did not have a fever when admitted indicated that the absence of fever might not rule out the existence of COVID-19. A considerable number of patients without fever could have been neglected if fever is used to instigate screening for COVID-19. It is noteworthy that only 85.2% of the deceased patients had a fever and that it was substantially less than those who managed to survive. These results demonstrate that patients with weak or inadequate responses to the virus are more vulnerable to a serious disease.

Cardiac complications are more common in pneumonia patients. Risk factors for post-pneumonia cardiac abnormalities usually involve advanced age, prior CVDs, and pneumonia with higher severity on presentation [21]. Acute cardiac events and adverse outcomes have been shown to cause cardiac abnormalities in influenza and some other respiratory viral infections [22]. Higher concentration in cardiac troponin I levels suggest a significantly higher risk of cardiac events such as heart failure or myocardial infarction [23,24]. In the current analysis, increased concentration in high-sensitivity cardiac troponin I was found in 93.2% of those who died. Moreover, nearly 95% of patients with elevated d-dimer levels are found closely associated with in-hospital mortality in our study. Previous studies have demonstrated that a higher proportion of increased d-dimer levels may be associated with the fatal consequence of SARS-CoV-2 infection [25,26]. LDH has been known as a proxy for severe prognosis in different diseases such as cancer and infection [27]. The increased level of LDH in SARS-CoV-19 patients indicated that LDH could be associated with lung disease and tissue damage, justifying a possible mechanism of investigation.

Though bacterial infections are generally thought to be the leading cause of sepsis, sepsis syndrome may also be caused by viral infection. Recently, a study in Wuhan revealed that almost 40% of adults with community-acquired viral pneumonia progressed with sepsis [28]. In another study, sepsis was reported as the most prevalent complication with 59% of the selected population [29]. In our study, almost half of the patients suffered septicemia. Besides, we observed that more than 50% of deceased patients had elevated WBC and PCT levels. Sepsis is a frequent complication that could be directly caused by infection with SARS-CoV-2, although it is important to carry out more studies on the pathogenesis of sepsis in COVID-19. The proportion of developing ARDS among deceased patients was higher as compared with survivors. As no appropriate antiviral medicines for SARS-CoV-2 are presently available, timely diagnosis and early respiratory assistance can deliver relief and mitigate mortality. The severity of illness in patients with preliminary positive nucleic acid test outcome was comparable to that of all patients with COVID-19. To minimize the risk of death in COVID-19 patients, as well as the risk of severe complications, urgent public health measures should be properly planned and implemented on those vulnerable populations. To detect early manifestations of clinical problems, thorough and regular follow-up is warranted.

Interestingly, about 13% of infected patients were vaccinated, suggesting that COVID-19 vaccinations are not 100% efficient at preventing infection. Vaccine breakthrough infections are likely to occur. People who are fully vaccinated and develop COVID-19 are less likely to acquire severe illnesses than patients who are unvaccinated and infect COVID-19. Nonetheless, unvaccinated individuals have a considerably higher risk of illness than those who have been vaccinated. According to studies, vaccinated individuals are 8 times less likely to become infected and 25 times less likely to be hospitalized or die. Vaccinations continue to prevent the majority of individuals from COVID-19 infection and associated consequences [30].

There are some limitations to the present study. To begin with, not all lab tests were made in all patients because of their retrospective study structure. Consequently, their role in predicting in-hospital mortality may be underestimated. Second, the lack of evidence on certain variables might contribute to an error in the assessment and minimize the representativeness of the sample. Thirdly, the case fatality ratio in our sample may not truly represent the mortality from COVID-19, due to the elimination of patients who were still hospitalized as of 10 May 2021, and therefore comparatively more severely ill at an early stage. Fourth, patients have sometimes been admitted late during their illness to the designated hospital. Failure to administer antivirals efficiently, poor compliance with standard supportive care, and high dosage corticosteroid use may also have led to adverse clinical outcomes in a few cases. Finally, yet importantly, the explanation of the given results may be constrained by the representative sample. However, we assume that the selected sample population is illustrative of cases identified and treated in only Islamabad by incorporating all patients in one designated COVID-19 hospital.

## 5. Conclusions

To the best of the author’s knowledge, this may be the largest comprehensive retrospective cohort study among SARS-CoV-2 patients who have encountered a definite outcome in the Pakistani population. We reported that old age, hypertension, pre-existing CVD, increased concentration of cardiac troponin I, and d-dimer on admission were autonomous risk factors for in-hospital death of COVID-19 patients. An additional consideration is needed to be given to these significant variables and further research on the fundamental mechanisms of these impacts. In conclusion, an in-depth study is still needed for patients with COVID-19. Reliable, rapid pathogen detection and practical differential diagnosis based on the clinical description are crucial for clinicians to contact suspected patients for the first time. Large longitudinal studies with the comprehensive analysis of all associated risk factors and possible confounders, as well as the evaluation of the treatment on progression and the prognosis of COVID-19, are warranted.

Moreover, the statistics provided in the present research could conceivably be utilized as an epidemiological viral and host attribute reference for further coronavirus outbreaks or, probably, another epidemic causative agent, assisting health authorities in identifying individuals at risk and integrating disease management efforts.

## Figures and Tables

**Table 1:** Demographic and clinical characteristics of COVID-19 patients and in-hospital outcomes.

	Frequency *n* (%)	Discharged *n* (%)	Deceased *n* (%)	*p*-Value
Age (years)	56 (18–88)	54 (46–63)	65 (58–88)	0.000
~50	125 (6.1)	77 (5.3)	48 (8.2)	
51–60	242 (11.8)	135 (9.3)	107 (18.0)	
61–70	734 (35.8)	570 (39.1)	164 (27.9)	
71~	947 (46.2)	676 (46.4)	271 (45.9)	
Sex				0.102
Female	831 (40.6)	656 (45.0)	175 (29.7)	
Male	1217 (59.4)	802 (55.0)	415 (70.3)	
Obese (BMI ≥ 30)	1468 (71.7)	1265 (86.8)	203 (34.4)	0.026
Smoker	522 (25.5)	416 (28.5)	106 (18.0)	0.050
Exposure history	1613 (78.8)	1420 (97.4)	193 (32.8)	0.861
Time from illness onset to outpatient visit, (d)	2 (1–5)	2 (1–4)	3 (1–6)	0.011
Time from illness onset to hospitalization, (d)	12 (8–15)	11 (8–14)	12 (9–16)	0.024
Symptoms at the time of admission, n (%)				
Fever (temp ≥ 37 °C)	1941 (94.8)	1439 (98.7)	502 (85.1)	0.001
Cough	1486 (72.6)	1061 (72.8)	425 (72.0)	0.659
Fatigue	1177 (57.5)	781 (53.6)	396 (67.1)	0.316
Sore throat	1167 (57.0)	781 (53.6)	386 (65.4)	0.681
Chest pain	1120 (54.7)	850 (58.3)	270 (45.8)	0.121
Nasal congestion/rhinorrhea	1034 (50.5)	858 (58.9)	176 (29.8)	0.551
Dyspnea	946 (46.2)	637 (43.7)	309 (52.4)	0.332
Chill	946 (46.2)	570 (39.1)	376 (63.7)	0.835
Dizziness and headache	686 (33.5)	396 (27.2)	290 (49.2)	0.054
Urinary incontinency	626 (30.6)	521 (35.8)	105 (17.8)	0.724
Chest distress	598 (29.2)	482 (33.1)	116 (19.7)	0.011
Abdominal pain	409 (20.0)	319 (21.9)	90 (15.3)	0.933
Myalgia or arthralgia	309 (15.1)	202 (13.9)	107 (18.1)	0.021
Diarrhea	202 (9.9)	87 (6.0)	115 (19.5)	0.431
Nausea and vomiting	125 (6.1)	48 (3.3)	77 (13.1)	0.657
Comorbidities, n (%)				
CVDs	1177 (57.5)	975 (66.9)	202 (34.2)	0.000
Hypertension	975 (47.6)	531 (36.4)	444 (75.3)	0.000
Cerebrovascular diseases	629 (30.7)	319 (21.9)	310 (52.5)	0.423
DM	608 (29.7)	213 (14.6)	395 (66.9)	0.001
COPD	154 (7.5)	88 (6.0)	66 (11.2)	0.132
Asthma	135 (6.6)	58 (4.0)	77 (13.1)	0.010
Tuberculosis	96 (4.7)	48 (3.3)	48 (8.1)	0.067
Chronic liver disease	79 (3.8)	38 (2.6)	41 (6.9)	0.042
Chronic renal disease	68 (3.3)	48 (3.3)	20 (3.4)	0.044
Cancer	50 (2.4)	29 (2.0)	21 (3.6)	0.452
HIV	29 (1.4)	19 (1.3)	10 (1.7)	0.547
Severity assessment on admission				0.002
Non-severe	637 (31.1)	483 (33.1)	154 (26.1)	
Severe	1411 (68.9)	1138 (78.1)	273 (46.3)	

Abbreviations: BMI: body mass index, CVDs: cardiovascular diseases, DM: Diabetes Mellitus, COPD: chronic obstructive pulmonary disease, HIV: human immunodeficiency virus, COVID-19: coronavirus 2019, *p* values comparing survivors and deaths of patients were obtained from chi-square test, Fisher exact test, or Mann–Whitney U test.

**Table 2:** Laboratory and radiological indicators of COVID-19 patients and in-hospital outcome.

	Frequency *n* (%)	Discharged *n* (%)	Deceased *n* (%)	*p*-Value
Laboratory indicators, *n* (%)	655 (32.0)	424 (29.1)	231 (39.2)	0.005
Higher neutrophil count (×10^9^/L)	1169 (57.1)	627 (43.0)	542 (91.9)	0.000
Lower lymphocyte count (×10^9^/L)	590 (28.8)	280 (19.2)	310 (52.5)	0.863
Higher leukocyte count (×10^9^/L)	696 (34.0)	338 (23.2)	358 (60.7)	0.471
Lower platelet count (×10⁹/L)	1081 (52.8)	531 (36.4)	550 (93.2)	0.000
Elevated cardiac troponin I, ng/mL	320 (15.6)	155 (10.6)	165 (27.9)	0.002
Increased CK (>185 U/L)	754 (36.8)	357 (24.5)	397 (67.3)	0.004
High LDH (>250 U/L)	686 (33.5)	397 (27.2)	289 (49.1)	0.093
Elevated ALT (>45 IU/L)	629 (30.7)	319 (21.9)	310 (52.5)	0.631
Elevated AST (>45 IU/L)	522 (25.5)	376 (25.8)	146 (24.7)	0.841
Higher creatinine (>1.3 mg/dL)	975 (47.6)	579 (39.7)	396 (67.1)	0.334
Albumin ≤ 35 (g/L)	860 (42.0)	608 (41.7)	252 (42.7)	0.451
Globulin > 85 (g/L)	590 (28.8)	280 (19.2)	310 (52.5)	0.001
Increased PCT	1806 (88.2)	1304 (89.4)	502 (85.1)	0.663
Increased CRP	821 (40.1)	328 (22.5)	493 (83.6)	0.821
ESR (>20 mm/h)	445 (21.7)	193 (13.2)	252 (42.7)	0.000
High serum ferritin level (>300 µg/L)	1785 (87.2)	1264 (86.7)	521 (88.3)	0.000
IL-6 > 7 ng/L	502 (24.5)	232 (15.9)	270 (45.8)	0.237
IL-8 > 62 ng/L	899 (43.9)	435 (29.8)	464 (78.6)	0.065
TNF-α > 8.1 ng/L	1575 (76.9)	1245 (85.4)	330 (55.9)	0.289
Proteinuria	975 (47.6)	416 (28.5)	559 (94.7)	0.000
Increased D-dimer > 1µg/L	145 (7.1)	77 (5.3)	68 (11.5)	0.303
Prolonged thrombin time				0.005
Imaging features	1169 (57.1)	695 (47.7)	474 (80.3)	
Bilateral distribution, *n* (%)	522 (25.5)	125 (8.6)	397 (67.2)	
Pleural effusion, *n* (%)	655 (32.0)	424 (29.1)	231 (39.2)	0.005

Abbreviations: CK: creatinine kinase, ALT: alanine transaminase, AST: aspartate transaminase, PCT: procalcitonin, CRP: c-reactive protein, ESR: erythrocyte sedimentation rate, LDH: Lactate dehydrogenase, *p* values comparing survivors and deaths of patients were obtained from chi-square test, Fisher exact test, or Mann–Whitney U test.

**Table 3:** Complications and treatment during hospitalization for COVID-19 patients and in-hospital outcome.

	Frequency *n* (%)	Discharged *n* (%)	Deceased *n* (%)	*p*-Value
Complications				
Septicemia	1062 (51.9)	502 (34.4)	560 (94.9)	0.000
ARDS	975 (47.6)	397 (27.2)	578 (98.0)	0.000
Respiratory failure	850 (41.5)	280 (19.2)	570 (96.6)	0.000
Acute kidney injury	367 (17.9)	29 (2.0)	338 (57.3)	0.225
Acute cardiac injury	395 (19.3)	15 (1.0)	380 (64.4)	0.000
Liver dysfunction	522 (25.5)	222 (15.2)	300 (50.8)	0.872
Coagulopathy	406 (19.8)	106 (7.3)	300 (50.8)	0.423
Bacteremia	358 (17.5)	184 (12.6)	174 (29.5)	0.060
Hyperglycemia	1331 (30.7)	808 (55.4)	523 (88.6)	0.001
Acidosis	205 (10.0)	87 (6.0)	118 (20.0)	0.478
Treatment				
Antibiotics	1970 (96.2)	1381 (94.7)	589 (99.8)	0.022
Antiviral drugs	502 (24.5)	233 (16.0)	269 (45.6)	0.032
Intravenous corticosteroid	627 (30.6)	328 (22.5)	299 (50.7)	0.475
Intravenous immunoglobulin	530 (25.9)	165 (11.3)	365 (61.9)	0.012
Oxygen therapy				0.000
High flow oxygen therapy	395 (19.3)	96 (6.6)	299 (50.7)	
Nasal cannula or mask	377 (18.4)	106 (7.3)	271 (45.9)	
Noninvasive mechanical ventilation	291 (14.2)	29 (2.0)	262 (44.4)	
Invasive mechanical ventilation	737 (36.0)	165 (11.3)	572 (96.9)	
ECMO	68 (3.3)	15 (1.0)	53 (9.0)	0.020
Continuous Renal replacement therapy	174 (8.5)	15 (1.0)	159 (26.9)	0.000

Abbreviations: ARDS: acute respiratory distress syndrome, ECMO: Extracorporeal membrane oxygenation, COVID-19: coronavirus 2019, *p* values comparing survivors and deaths of patients were obtained from chi-square test, Fisher exact test, or Mann–Whitney U test.

**Table 4:** Multivariate analysis of risk factors associated with COVID-19 patients.

	Regression Coefficient (95% CI)	*p*-Value
Age	1.121 (0.974–2.131)	0.000
Fever	1.302 (1.126–2.032)	0.124
Hypertension	2.634 (2.132–3.131)	0.001
Diabetes	2.851 (1.565–3.325)	0.055
CVD	2.043 (1.965–2.456)	0.001
Higher neutrophil count (×10^9^/L)	0.788 (0.374–1.212)	0.131
Lower lymphocyte count (×10^9^/L)	−0.620 (−0.830–0.004)	0.052
Elevated cardiac troponin I, ng/mL	2.133 (1.067–2.541)	0.000
High LDH	0.861 (0.473–1.033)	0.051
Increased CK (>185 U/L)	0.412 (0.101–1.001)	0.330
Increased PCT	0.023 (0.003–0.063)	0.228
High serum ferritin level (>300 µg/L)	1.006 (0.937–1.073)	0.144
Increased D-dimer (>1 µg/L)	1.411 (1.226–2.161)	0.001
IL-6 > 7 ng/L	0.056 (0.009–0.125)	0.236
Septicemia	0.119 (0.106–0.328)	0.002
ARDS	1.236 (1.110–1.409)	0.001
Respiratory failure	1.070 (0.968–1.114)	0.165
Acute cardiac injury	1.339 (0.997–1.581)	0.065
Hyperglycemia	1.12 (1.074–1.241)	0.078
Bilateral distribution, *n* (%)	−0.050 (−0.089–0.456)	0.543

Abbreviation: CVD: cardiovascular disease, CK: Creatine kinase, ARDS: acute respiratory distress syndrome, LDH: lactate dehydrogenase.

## Data Availability

The datasets used and/or analyzed during the current study are available from the corresponding author on reasonable request.
